# Using Geosocial Networking Apps to Understand the Spatial Distribution of Gay and Bisexual Men: Pilot Study

**DOI:** 10.2196/publichealth.8931

**Published:** 2018-08-08

**Authors:** Kiffer George Card, Jeremy Gibbs, Nathan John Lachowsky, Blake W Hawkins, Miranda Compton, Joshua Edward, Travis Salway, Maya K Gislason, Robert S Hogg

**Affiliations:** ^1^ School of Public Health and Social Policy Faculty of Human and Social Development University of Victoria Victoria, BC Canada; ^2^ School of Social Work University of Georgia Athens, GA United States; ^3^ University of British Columbia Vancouver, BC Canada; ^4^ Vancouver Coastal Health Vancouver, BC Canada; ^5^ Health Initiative for Men Vancouver, BC Canada; ^6^ Community Based Research Centre for Gay Men’s Health Vancouver, BC Canada; ^7^ Faculty of Health Sciences Simon Fraser University Burnaby, BC Canada

**Keywords:** service access, geosocial networking apps, gay and bisexual men, spatial distribution, gay neighborhoods

## Abstract

**Background:**

While services tailored for gay, bisexual, and other men who have sex with men (gbMSM) may provide support for this vulnerable population, planning access to these services can be difficult due to the unknown spatial distribution of gbMSM outside of gay-centered neighborhoods. This is particularly true since the emergence of geosocial networking apps, which have become a widely used venue for meeting sexual partners.

**Objective:**

The goal of our research was to estimate the spatial density of app users across Metro Vancouver and identify the independent and adjusted neighborhood-level factors that predict app user density.

**Methods:**

This pilot study used a popular geosocial networking app to estimate the spatial density of app users across rural and urban Metro Vancouver. Multiple Poisson regression models were then constructed to model the relationship between app user density and areal population-weighted neighbourhood-level factors from the 2016 Canadian Census and National Household Survey.

**Results:**

A total of 2021 app user profiles were counted within 1 mile of 263 sampling locations. In a multivariate model controlling for time of day, app user density was associated with several dissemination area–level characteristics, including population density (per 100; incidence rate ratio [IRR] 1.03, 95% CI 1.02-1.04), average household size (IRR 0.26, 95% CI 0.11-0.62), average age of males (IRR 0.93, 95% CI 0.88-0.98), median income of males (IRR 0.96, 95% CI 0.92-0.99), proportion of males who were not married (IRR 1.08, 95% CI 1.02-1.13), proportion of males with a postsecondary education (IRR 1.06, 95% CI 1.03-1.10), proportion of males who are immigrants (IRR 1.04, 95% CI 1.004-1.07), and proportion of males living below the low-income cutoff level (IRR 0.93, 95% CI 0.89-0.98).

**Conclusions:**

This pilot study demonstrates how the combination of geosocial networking apps and administrative datasets might help care providers, planners, and community leaders target online and offline interventions for gbMSM who use apps.

## Introduction

In British Columbia, Canada, HIV and other sexually transmitted infections continue to disproportionately impact gay, bisexual, and other men who have sex with men (gbMSM) [1,2]. Yet, because the spatial geography of gbMSM may not correlate with that of the broader population, it remains difficult to ensure that sexual health and other services are optimally tailored for these individuals [3]. Previous research examining the social geography of gbMSM has shown that their spatial distribution is nonrandom [4] within the general population. For example, research suggests that the marginalization of sexual minorities along with other forces has given rise to gay neighborhoods—areas that often have a higher than expected concentration of gay men, gay-centered amenities, and homonormative cultural artifacts [5]. However, changing attitudes toward gbMSM in Western society have supposedly reshaped these communities, leading to changes in where these men live, work, and socialize [6]. Additionally, current literature indicates that the introduction of geosocial networking apps, which allow gbMSM to use smart devices to connect with other gbMSM within their geographic proximity, has reduced the need for traditional gay enclaves to facilitate connection [7,8]. These changes challenge the assumption that sexual health services tailored for gbMSM are only needed (or appropriate) within these historically gay neighborhoods [9]. Further compounding the difficulty of targeting app users, their spatial geography may not correlate with that of the wider gbMSM population. For example, previous research has found that only 10% of rural gbMSM sought sex online, compared with 56% in medium sized cities, 50% in suburban areas, and 48% in urban centers [10]. However, dating and online hookup apps largely appeared on the scene in 2009, after this research was conducted; therefore, it is unclear whether these patterns hold true today. These realities make it difficult to identify where and how sexual health services can best meet the needs of app users who are at elevated risk for HIV and other sexually transmitted infections.

Methods in examining app user density have not been widely explored. This study is the first of its kind in Canada and is only preceded by the work of Delaney et al [3], who used similar methods in estimating app user density in Atlanta, Georgia. In their pilot, the authors used a geosocial networking app designed for gbMSM to manually sample 2666 app users across 79 sampling locations. Sampling locations were selected by starting at the home of 1 of the researchers and driving along road networks to create 2-mile sampling intervals throughout the city. In areas where app user density was greater than 50 users per 2-mile radius, they recorded the maximum distance to the 50th closest user and traveled to the next sampling point outside of that buffer. This sampling strategy resulted in 79 data collection points across the city, many of which overlapped. The data were then smoothed using ArcGIS’s kernel density tool (Esri) [11] to create a density map of app users. While Delaney’s objectives were primarily descriptive, our study seeks to modify and leverage their sampling methods to estimate the spatial density of app users across Metro Vancouver and identify the independent and adjusted neighborhood-level factors that predict app user density. The latter of these 2 objectives has not yet been explored despite studies in other research contexts suggesting that neighborhood-level factors are related to the health and behavior of gbMSM [12,13].

## Methods

### Study Setting

**Figure 1:**
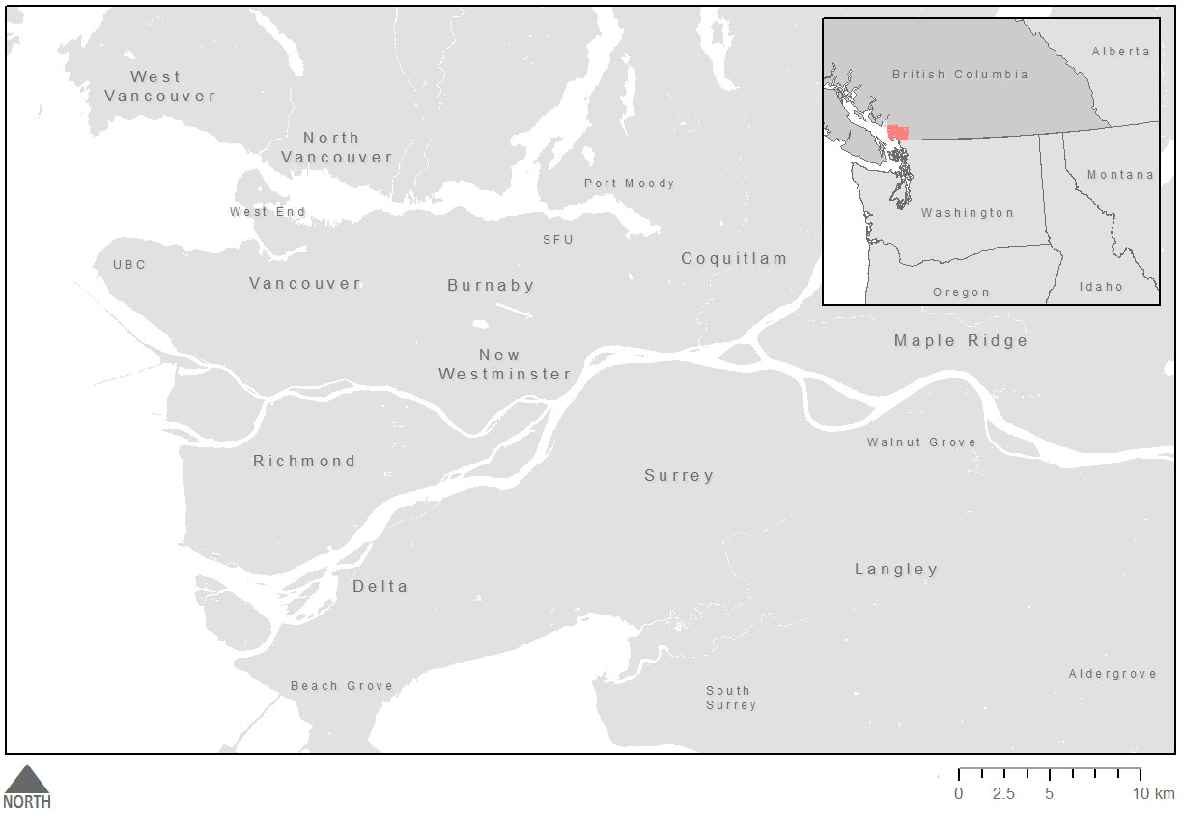
Study setting.

This pilot study took place in Metro Vancouver, a regional district of British Columbia, Canada (see Figure 1). Metro Vancouver is a favorable location for examining the delivery of sexual health services as it offers a highly supportive environment for sexual minorities and for people living with HIV [14-16]. Since the late 1990s, the province has provided HIV medications and testing services free of charge, with much of the HIV treatment services being administered centrally by the British Columbia Centre for Excellence in HIV/AIDS [16]. Further, the province has led the way in several global initiatives, including the Joint United Nations Programme on HIV/AIDS 90-90-90 worldwide strategy for HIV prevention [17]. Further, Metro Vancouver is an ideal location to consider app use and the spatial variation in gender and sexual minority populations, as it has an active lesbian, gay, bisexual, and transgender (LGBT) community, evidenced by its hosting of an annual gay pride parade, several community-based organizations for lesbian, gay, bisexual, transgender, and queer people, gay bathhouses and bars, and other attractive amenities. Many of these attractions are in the downtown West End (Vancouver’s historically gay neighborhood), however smaller municipalities such as New Westminster are also home to gay bathhouses and gay-owned businesses.

### Data Collection

#### App User Density

**Figure 2:**
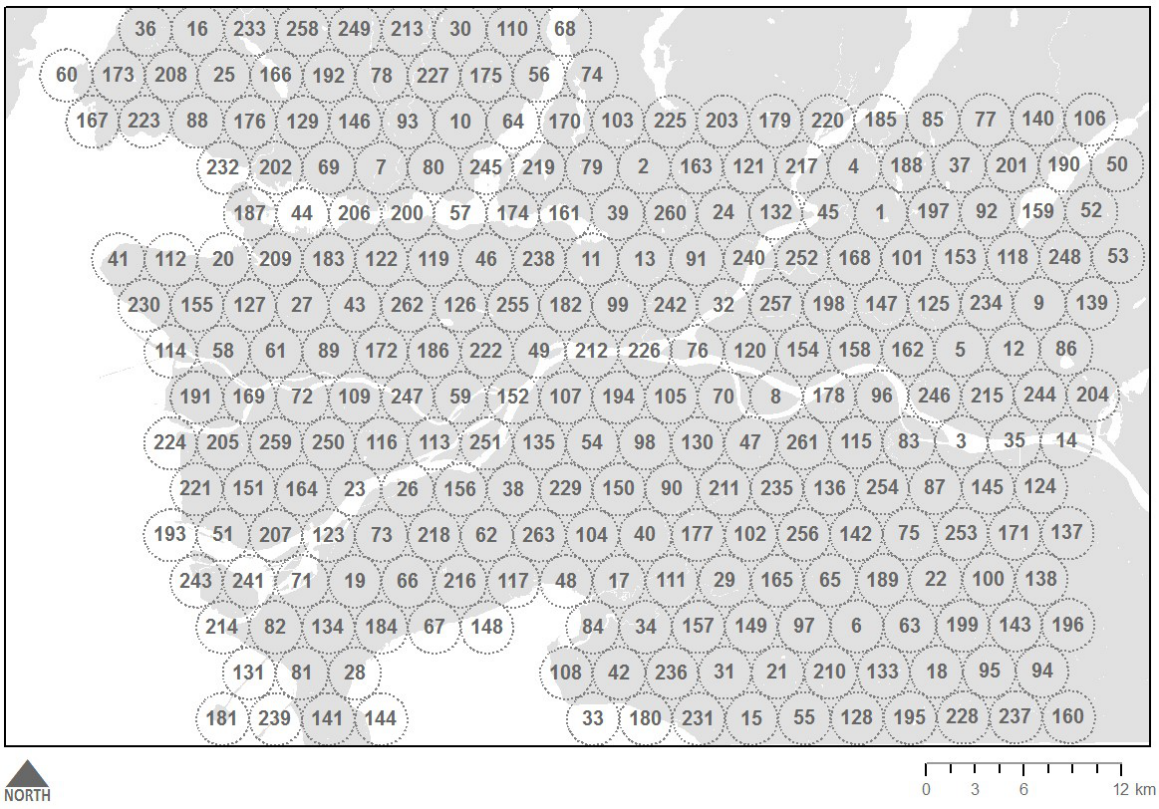
Sampling strategy for mapping app user density. Dotted line represents 1-mile radius around each sampling location. Numbers represent the order in which location was sampled.

Like Delaney et al [3], we used a popular geosocial networking app designed for gbMSM and primarily used by people looking for casual sexual partners, dates, or relationships [7]. While several similar apps exist—targeting a wide range of gbMSM subgroups—the app selected for our study was chosen because it is among the most popular apps for gbMSM [18]. When creating or editing their profile, users of this app can elect to provide a picture and headline for their profile, which is displayed in a grid alongside other users, organized by increasing Euclidian distance [19]. Only active or recently active (ie, within 1 hour) profiles are displayed. Tapping on each photo reveals volunteered information, composing a user’s profile. Further, and of greatest relevance to this study, users are also asked whether they would like to grant access to their location data, which in turn is displayed to other users as real-time Euclidian distance [19]. We should note that the app used in this pilot study is not necessarily representative of all apps used by gbMSM, and we expect that future analyses will explore and compare the results from available platforms. Nevertheless, using this platform, we modified Delaney’s data collection method by systematically sampling app users across a grid of predetermined data collection points throughout Metro Vancouver (see Figure 2). The first collection point was selected randomly from a location in Metro Vancouver, and the grid was created by calculating the coordinates for points at 2-mile intervals. Rather than physically traversing the city, as in Delaney et al [3], this approach allowed us to estimate app user density by putting the coordinates of each sampling location into our phone and then counting the number of profiles within a 1-mile radius of each sampling location. This distance was chosen because the app allows users to see the distance (in feet) of other app users up to a 1-mile radius, beyond which the distance of other users is measured with less precision (in miles). As we were only counting the number of users within each sampling radii, no data were collected from user profiles. Collection of other profile data was avoided as an extra precaution beyond traditional ethics guidelines due to the need for further ethical guidance on the use of internet-embedded, publicly available geotagged data for public health and research purposes [20].

As some users did not display their location on their profile, we did not count users who withheld their location and were listed on our screen such that it was unclear whether they were within 1 mile of our virtual sampling location (although we did count users without location information when their inclusion was unambiguous). Recognizing that the desire for greater privacy might vary spatially, this limitation has the potential to underestimate the number of users at some sampling locations (eg, where discreet users worry that they might be identified based on their location). In evaluating the extent to which this limitation impacted our results, we sampled 500 profiles across 5 spatially diverse sampling locations and found that 25.4% (127/500, range 19 to 32) of users did not provide location information. Of these, 5.5% (7/127, range 0 to 3) were listed such that their privacy settings made their inclusion ambiguous (ie, less or greater than 1 mile). The remaining 120 participants did not provide location information but were listed such that dichotomizing their location (eg, 1 mile or more, less than 1 mile) was not difficult (ie, they appeared earlier in the distance-ordered list of users than the farthest participant within 1 mile, thus indicating they resided within 1 mile).

As previous research has shown that app use is higher in the evening and on weekdays [21], data were collected between 5:45 pm and 11:00 pm, Monday through Wednesday, in the last week of November 2016. Dates were selected to represent a normal weekday (eg, no holidays or LGBT events). To further control for variance in use across time (ie, peak hours), we used a random number generator to randomize the order in which geographic locations were sampled. As users can access apps from anywhere (eg, work, home, bars, bathhouse), it is likely that some users access the app from multiple locations throughout their day or week; therefore, individuals were blocked so that they were not counted multiple times. When accessing the app platform, we used a blank profile and did not respond to private messages.

#### Neighborhood Factors

Recognizing that social and demographic factors have previously been associated with app use [22-25], risky sexual behavior [4,26-29], and neighborhood residence among gay and bisexual men [28,30-32], selected sociodemographic variables for each dissemination area were derived from the 2016 Canadian Census using the Census Analyzer developed by Computing in the Humanities and Social Sciences at the University of Toronto. Additional information on this data source is available elsewhere [33]. Brief definitions for each variable included in our study are provided in Textbox 1. Selection of included variables was made based on their ubiquity in administrative datasets and scientific surveys, thus improving the reproducibility of our study [34]. Furthermore, measuring urbanity, gender, age, ethnicity, socioeconomic status, family situation, and immigration status, the selected variables represented a variety of factors which have regularly been associated with health-related outcomes [35-40].

### Statistical Analysis

Spatial data were generated in ArcMap version 10.5 (Esri), and statistical modeling was conducted in R version 3.4.4 (The R Foundation). Bivariate and multivariable Poisson regression models were used to identify neighborhood-level factors associated with greater app user density. The spatial unit of analysis for this regression was the 1-mile sampling radius around each virtual sampling point. For each unit, app user density, rounded to the nearest integer, was calculated by dividing the number of app users observed at each sampling location by the land area within the 1-mile sampling radius. As explanatory variables were on the dissemination area level, we created a combined area and population-weighted average for each factor, which took into account the population size of each dissemination area as well as the proportion of the dissemination area within each sampling radius [41]. Final multivariable models were constructed by initially including all candidate variables of interest and then optimizing the Akaike information criterion (AIC) by backwards elimination. As our sampling method may have biased the app user density of location, we forced inclusion of an interaction term that controlled for time of day (ie, before 8 pm, 8 pm or later) and day of week (ie, Monday, Tuesday, or Wednesday). As a widely used variable selection method [42], particularly for exploratory analyses such as those conducted in our study, this backwards elimination procedure allowed us to identify the relatively best fitting statistical model achievable from our candidate variables, thus simultaneously improving the reproducibility of our study procedures and ensuring the optimal inclusion of candidate variables under conditions where closely related measures (eg, income and education) might limit model accuracy or performance. Comparing the final multivariable model to 1 including only population density and our time-day interaction term, we used a likelihood ratio test [43] and a Bonferroni outlier test [44], the latter of which allowed us to assess the relative performance of the models and detect geographic areas of interest with statistically unexpected app user densities.

**Definitions of census dissemination area level characteristics.**

- Population density (per 100): total population of all persons living in each dissemination area divided by the land area of the dissemination area. Modeled as a per 100 resident increase in persons per km^2^.
- Percentage of residents who are male: percentage of residents in each dissemination area who are male.
- Average age of male residents: average age of male residents in each dissemination area.
- Median income of male residents (per Can $1000 [US $1300]): median annual income of male residents in each dissemination area. Modeled as per Can $1000 increase in annual income.
- Percentage of male residents not married: percentage of male residents in each dissemination area who were not married and not living with a common-law partner, including those who were never married, separated, divorced, or widowed.
- Percentage of male residents with a postsecondary education: percentage of male residents in each dissemination area who have credentials beyond that of a high school diploma, including trade and apprenticeship certificates, college degrees, and university degrees.
- Percentage of male residents living below the low income cutoff (LICO) level: proportion of male residents in each dissemination area living below the Canadian Census Bureau’s LICO level (ie, those with after-tax income levels more than 20 percentage points below that required to afford food, shelter, and clothing in the dissemination area in which they reside).
- Percentage of males who are unemployed: percentage of male residents in each dissemination area who are unemployed.
- Percentage of male residents who are immigrants: percentage of male residents in each dissemination area who were born outside of Canada.
- Percentage of male residents who are visible minorities: percentage of male residents in each dissemination who are non-Caucasian in race or nonwhite in color and who are not indigenous.
- Average household size of residents: average number of persons who occupy the same dwelling unit and do not have a usual place of residence elsewhere in Canada or abroad.

Model fit was assessed using the McFadden likelihood-based pseudo *r*^2^ and by reviewing other postmodel evaluation criteria (such as the distributions of residuals). The Office of Research Ethics at Simon Fraser University waived ethics approval, as we collected only publicly accessible data (ie, counted the number of profiles near each sampling location) and did not engage users.

## Results

**Figure 3:**
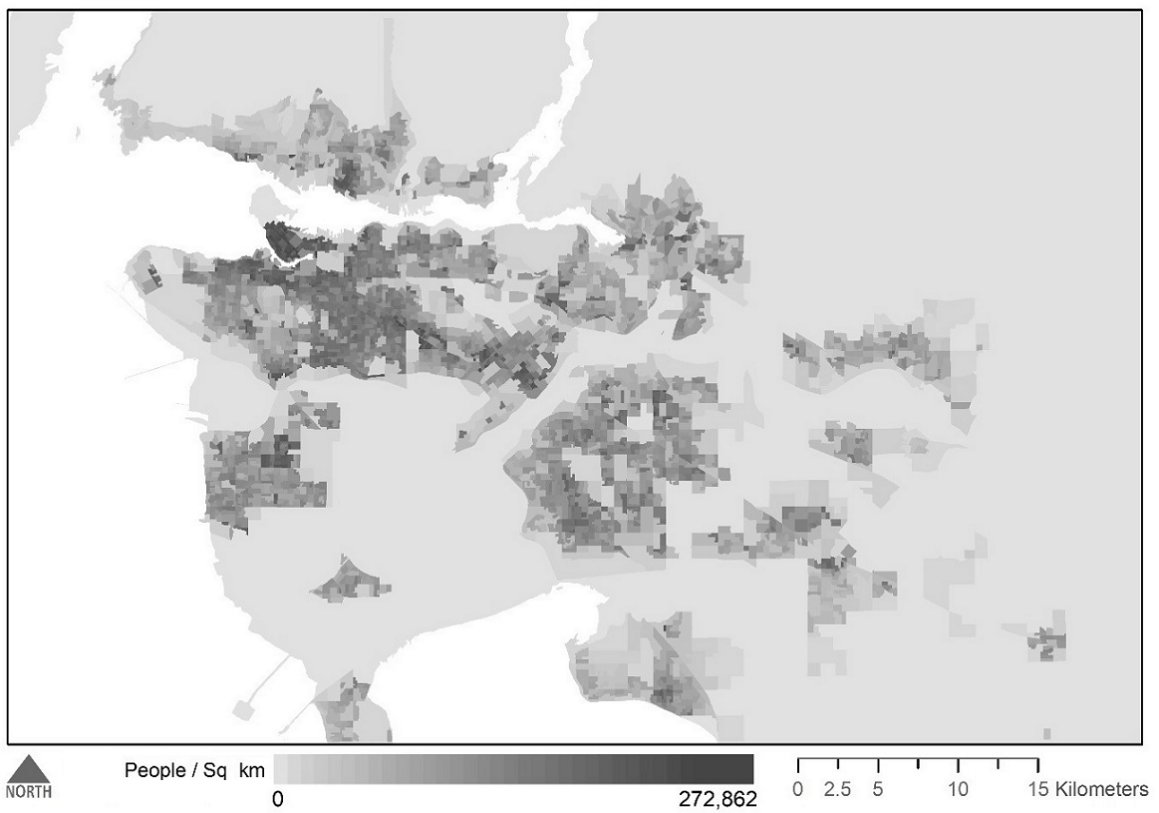
Population density of dissemination areas in Metro Vancouver, colored by quantiles.

**Figure 4:**
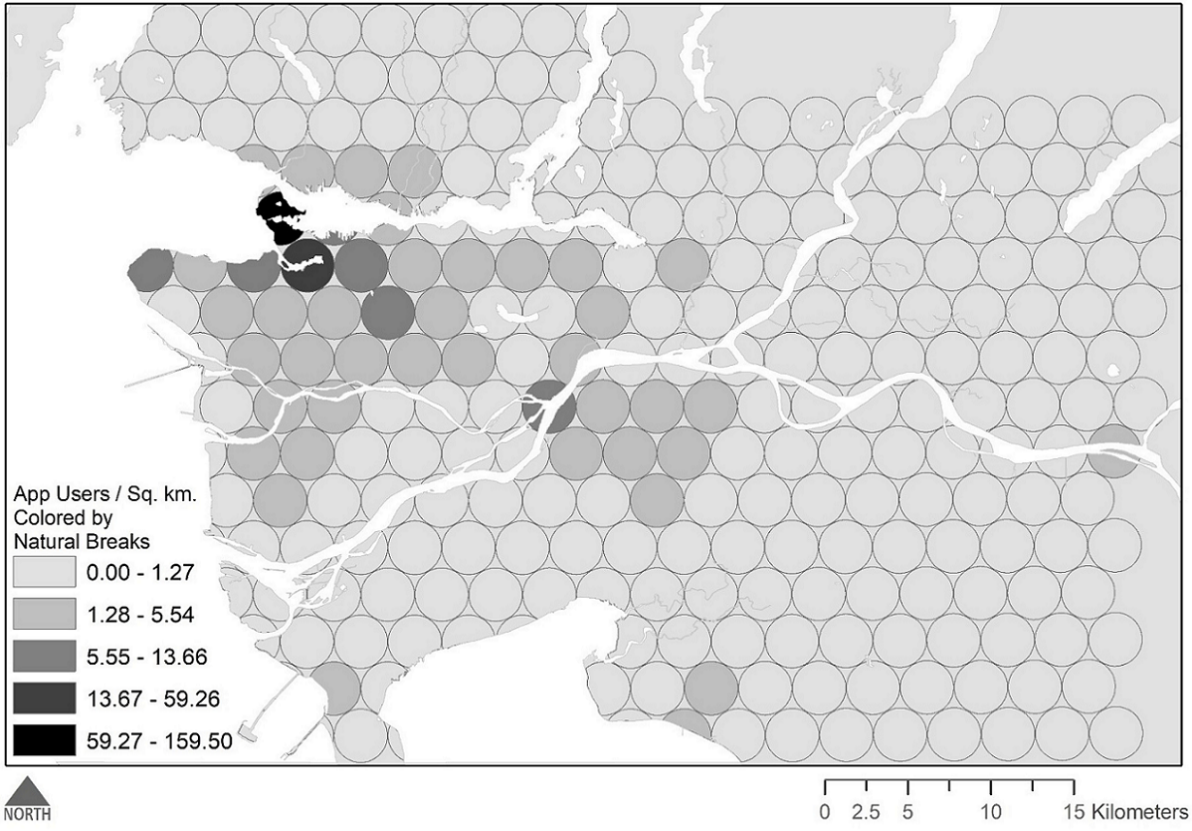
Observed density of app users, colored by natural breaks.

A total of 2021 app user profiles were counted within 1 mile of 263 sampling locations. Figure 3 presents the population density of each dissemination area, and Figure 4 presents the observed app user densities at each sampling buffer. Table 1 provides descriptive statistics for each dissemination area–level characteristic examined in our model and the bivariate associations with app user density.

**Figure 5:**
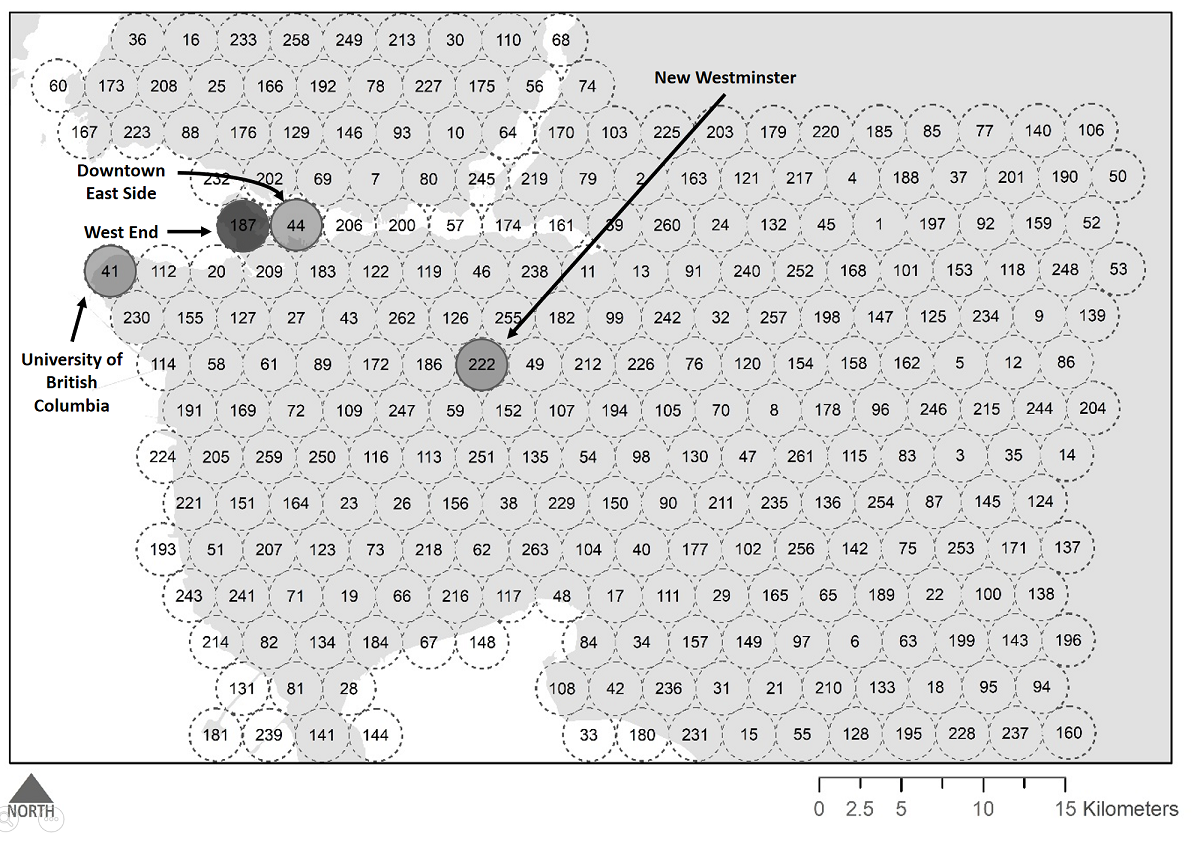
Model outliers in population density–only model (light and dark gray) and final multivariate model (dark gray only).

In our simplified model examining the association between app user density and population density (controlling for time and day of sampling), each 100-person increase in population density was associated with a 6.2% increase in app user density (incidence rate ratio [IRR] 1.06, 95% CI 1.06-1.07). As suggested by an increase in model fit (pseudo *r*^2^ .650 to .760), the results of a likelihood ratio test (*P*<.001), and a 4-fold reduction in the number of outliers (Figure 5) identified by a Bonferroni model outlier test (ie, 4 to 1), an AIC optimized model including all dissemination area characteristics of interest had superior performance relative to this population density–only model.

As shown in Table 2, this expanded model showed that app user density was positively associated with population density, average age of male residents, proportion of male residents who were not married, proportion of males with a postsecondary education, proportion of male residents who were immigrants, proportion of males living below the low income cutoff (LICO) level, and average household size of residents.

**Table 1:** Descriptive statistics and bivariate associations with app user density for areal population-weighted dissemination area–level characteristics.

2016 Census variable	Median (Q1-Q3)	*P* value
Population density (persons/km^2^)	331.6 (59.2-1807.0)	<.001
Percentage of residents who are male	49.3 (48.6-50.5)	<.001
Average age of male residents (years)	41.1 (38.2-44.1)	.581
Median income of male residents (Can $)	48,567 (42,816-55,826)	<.001
Percentage of male residents not married	35.4 (30.9-40.5)	<.001
Percentage of male residents with a postsecondary education	57.6 (48.9-62.0)	<.001
Percentage of males who are unemployed	5.1 (3.4-6.1)	<.001
Percentage of male residents living below LICO^a^ level	7.0 (4.9-11.1)	<.001
Percentage of male residents who are immigrants	27.2 (18.4-38.8)	<.001
Percentage of male residents who are visible minorities	26.0 (12.4-46.8)	<.001
Average household size of residents	2.8 (2.6-3.0)	<.001

^a^LICO: low income cutoff.

**Table 2:** Multivariate Poisson regression examining areal population-weighted dissemination area–level characteristics associated with sampling area app user density.

Variable		Incidence rate ratio (95% CI)
Population density (per 100)		1.03 (1.02-1.04)
Average age of male residents		0.93 (0.88-0.98)
Median income of male residents		0.96 (0.92-0.99)
Percentage of male residents living below LICO^a^ level		0.93 (0.89-0.98)
Percentage of male residents with a postsecondary education		1.06 (1.03-1.10)
Percentage of male residents who are immigrants		1.04 (1.004-1.07)
Percentage of male residents not married		1.08 (1.02-1.13)
Average household size of residents		0.26 (0.11-0.62)
**Sampling time**		
	Monday: before 8:00 pm	Reference
	Monday: 8:00 pm or later	2.16 (1.24-3.83)
	Tuesday: before 8:00 pm	2.00 (1.07-3.79)
	Tuesday: 8:00 pm or later	2.28 (1.44-3.77)
	Wednesday: before 8:0 pm	1.15 (0.44-2.67)
	Wednesday: 8:00 pm or later	1.13 (0.67-1.94)

^a^LICO: low income cutoff.

## Discussion

### Principal Findings

Using a popular geosocial networking app designed for gbMSM, we sampled over 2000 profiles that were within 1 mile of 263 randomly selected sampling sites in Metro Vancouver, Canada. While our methodology extends those originally piloted by Delaney et al [3], this study is novel in its use of this approach to evaluate the relationship between app user density and other neighborhood-level factors. In doing so, this pilot study supports the use of geographic information systems in aiding public health specialists to understand the spatial distribution of app users. With that said, we acknowledge that the associations identified in our study may be the result of ecological fallacy. Addressing this possibility, we also recognize that several of the factors associated with app user density in this pilot study have also been shown to predict app use among gbMSM at the person level.

Beginning with the social geography of app use, we note that each 100-person increase in population density was associated with a 6% increase in app user density in unadjusted models and a 3% increase when accounting for other factors. Furthermore, we see in Figures 4 and 5 that app user density is dramatically higher in downtown Vancouver, particularly in the historically gay neighborhood of Davie Village. This, along with increased app user density in New Westminster (the location of several LGBT-friendly amenities including a gay bathhouse), shows that app user density tracks the distribution of other gay-centric amenities quite well, perhaps indicating that the social geography of online sex seeking has changed from the patterns observed earlier in the internet’s history, when online sex seekers were more likely to identify as bisexual, be closeted, live outside major urban centers, and be disconnected from the gay community [45]. If true, these patterns agree with recent community-based research among gbMSM in Metro Vancouver that suggests that online sex-seeking gbMSM actually spend more time with other gbMSM and are equally as likely to participate in the gay community compared with those who do not seek sex online [46]. With that said, these findings should not be interpreted to mean that rural gbMSM do not use online venues. To do so would be to conflate app use with app user density, the latter of which being a composite measure that includes both the spatial distribution of gbMSM and the prevalence of app use among these men. As such, we note that previous studies have shown that rural men rely on internet-enabled technologies to connect with one another, particularly in rural localities where gbMSM are stigmatized [47]. Interpreted with respect to this, it is possible that app user density is higher in urban areas due to both a preference among gbMSM to live in these areas [48] and the increased motivation for app use proffered by greater opportunities to meet nearby partners [49-51]. Regarding the first hypotheses, we should comment that a growing body of literature has come to question unidirectional migration patterns (ie, from rural to urban) of LGBT people [6,52,53], and research regarding the latter highlights how different motivations for technology use (eg, to meet nearby partners for casual sex) may motivate urban MSM to specifically use apps. With these varied perspectives in mind, we acknowledge that the relationship between online sex seeking, identity, disclosure, and community connectedness remain important areas of study for the health and social sciences [54].

More squarely within the focus of our pilot study, we found that each 1% increase in the proportion of males who were not married and each 1-person increase in average household size were associated with a respective 8% increase and 74% decrease in app user density. The opposing effects here are consistent on face value: with increasing household size being negatively associated with an increasing proportion of residents who are married. Likewise, given that previous research has shown that the technographics of online dating are heavily biased toward single and nonmonogamous users [22], an increasing proportion of single residents in a given neighborhood is expectedly associated with increasing app user density.

As with measures assessing marital status and household size, the observation that each 1-year increase in the average age of the male population was associated with a respective 7% decrease in app user density is unsurprising. Again, the technographics of app use tend to skew toward young gbMSM [46,55]. Thus, neighborhoods with a greater proportion of young men (and a lower average age) would be expected to have more app users. However, again referring to Figures 4 and 5, we can see that the outliers identified by our pilot study included the sampling area in which the University of British Columbia is located. Underscoring this spatial observation, we also documented a 6% increase in app user density for each 1% increase in the proportion of males who had a postsecondary education. This finding too is supported by recent person-level research in Metro Vancouver that has shown an association between greater educational attainment and online sex seeking [22]. Likewise, studies have documented higher educational attainment among adult sexual minorities [56]. Together, these disparate findings are suggestive of nuanced interrelationships between residential location, app use, educational attainment, and age. However, these cannot be fully explained by our findings here and require additional research regarding the life course of gay and bisexual men.

Moving to other closely related sociodemographic measures, our study found that each 1% increase in the proportion of males who were living below the LICO level and each Can $1000 (US $1300) increase in the median income of males were associated with a 7% and 4% decrease in app user density, respectively. As these associations present seemingly contradictory findings, we should first point out that median income and the proportion of residents living below the LICO threshold represent considerably different neighborhood and household conditions despite both serving as measures of socioeconomic status [57]. Median incomes are the median total income residents receive throughout a year. LICO thresholds are the income levels in each dissemination area below which a household would devote at least 20% more than the average family would on basic necessities (ie, food, clothing, and shelter) [58]. An increasing proportion of people living below LICO thresholds can indicate an increasing proportion of impoverished residents as well as an increasing cost of living in a given neighborhood. Therefore, the negative associations between app user density and these 2 measures may indicate that app user density is lower in both cash-strapped neighborhoods (regardless of overall income levels) and those where incomes are depressed. In either case, these trends may be associated with greater constraints placed on the time of residents or attributable to differing lifestyles of residents in these neighborhoods. Supporting this interpretation, previous research examining the association between individual income and app use found that app use on weekdays (during which this study was conducted) is associated with having lower income [21]. As such, caution should be taken when interpreting these findings, as patterns of app user density on weekends might eliminate or reverse this association. In any case, further qualitative research may be needed to understand how app use, neighborhood residence, and socioeconomic status relate to one another.

The same is likely true regarding the final measure included in our multivariable model. Indeed, as is often the case with research addressing multiple intersecting identities [59], to our knowledge little attention has been specifically devoted to the diverse phenomenon of app use among immigrant gbMSM or those living in semisegregated immigrant neighborhoods [60], yet in our study we found that each 1% increase in the proportion of males who were immigrants was associated with a 4% increase in app user density. It is possible that immigrants rely on apps as ways to connect with other gay men, perhaps due to the lack of LGBT venues available to them in ethnically segregated neighborhoods [61] or, alternatively, due to their desire to explore their sexuality discreetly [60]. In either case, this association highlights the importance of diversifying sexual health services and ensuring that they are accessible to those living outside traditional gay villages that often have the reputation of being for wealthy, white, gay men and their straight allies [62,63].

### Implications

Given the findings outlined, future studies are needed to assess the generalizability of these piloted methods and determine the generalizability of these results outside Metro Vancouver. Laying groundwork for such a validation, our pilot study provides a proof of concept for methods that might be used by public health leaders to optimize the delivery and focus of HIV prevention services by targeting populations at elevated risk for HIV transmission using administrative and geotagged data. While we are not aware of any studies that have leveraged this type of data to improve the delivery of HIV services (ie, location of new services, mobile testing vans) to high-risk neighborhoods, some work has shown that administrative data can be used to identify neighborhoods at risk for other adverse health outcomes [26]. Combining spatial data from various sources (such as dating apps) with administrative data may, therefore, provide an important opportunity for knowledge translation in the context of sexual health, allowing providers to deliver health care services to at-risk neighborhoods. This is especially true for jurisdictions that have invested in mobile testing services [64], online-initiated testing services [65], or other flexible health promotion programs. Further, by planning HIV care using a neighborhood-level perspective [66], public health and community leaders can better justify support for targeted interventions that can address the varied context-specific needs and concerns of local communities [4].

### Limitations

That said, the findings discussed are limited by several potential biases. First, and perhaps most importantly, readers should be aware that sociodemographic census-level factors may not reflect the characteristics of the app users sampled here. Second, because our explanatory variables are averaged across several dissemination areas, the accuracy of our estimates may be limited. However, because dissemination areas are administrative boundaries that are not necessarily reflective of the natural gradation of the characteristics, it is unclear to what extent these units might have biased our results. Future studies should employ a more purposeful sampling design that might better capture app user density within natural communities. Third, our data do not describe from where sampled users are accessing apps (eg, from bars or their home). Therefore, the data generated for this study do not necessarily reflect the residential location of gbMSM but rather where they use the apps on a typical weekday evening. Importantly, while the time and days selected for sampling were purposeful, the effects of sampling error may introduce bias into our study design. To account for this, we randomly assigned the order in which location points were sampled. However, it is still possible that temporal patterns of app use vary by some nonrandom factor (eg, daily routines). Indeed, it is not entirely clear how patterns of app use might vary across the day or week. Future analyses should explore these temporal patterns to determine why and to what degree app use varies across time and under what conditions gbMSM use apps. Fourth, this study was conducted using only a single app. While the app we selected is among the most popular apps for gbMSM [18], few studies have examined differences between apps that are targeted to and as a result taken up by specific subcultures or subgroups within the gay community. It is therefore possible that the spatial density of app users is reflective of only a subset of gbMSM who use apps to find sexual partners. Future work should investigate whether our results are reproducible with other apps such as those targeting older men, ethnic minority men, or men interested in “kink.” That said, previous research has shown that there is a large amount of overlap in the apps used by gbMSM. For instance, 1 study reported a median number of apps per user as 3.11 [21]. Fifth, as our multivariable model had a pseudo *r*^2^ of .76, omitted variables not accounted for in this study may also affect app user density. These likely include factors that are difficult to measure using administrative data or are at least rarely measured in these data sources, such as sexual orientation, prevalence of HIV, the social climate toward sexual minorities in a given neighborhood, or a person’s ability to meet sexual partners via other venues. Similarly, our models have yet to be validated for other settings and given that they were developed as exploratory, proof-of-concept models, further research is needed before these or similar models are used authoritatively to inform the deployment of health resources. Therefore, future studies should seek out other datasets and data sources from which models might be derived, thus providing a more complete and empirically valid picture of the ecological factors associated with app user density (eg, male population density vs general population density, same-sex households).

### Conclusions

Findings from this pilot study highlight the potential utility of using geographic information systems to better understand the spatial density of gbMSM, particularly among those who use geosocial networking apps and live in urban settings. While additional analyses are needed to validate the modeling techniques explored here and understand the impact of various sampling decisions (eg, time of day, choice of app provider), our findings suggest that these methods may be useful for public health and community leaders hoping to better understand the communities of gbMSM they serve.

## Abbreviations

AIC: Akaike information criterion
gbMSM: gay, bisexual, and other men who have sex with men
IRR: incidence rate ratio
LGBT: lesbian, gay, bisexual, and transgender
LICO: low income cutoff

## References

[1] (2014). British Columbia Provincial Health Officer.

[2] (2014). Public Health Agency of Canada.

[3] Delaney KP, Kramer MR, Waller LA, Flanders WD, Sullivan PS (2014). Using a geolocation social networking application to calculate the population density of sex-seeking gay men for research and prevention services. J Med Internet Res.

[4] Latkin CA, German D, Vlahov D, Galea S (2013). Neighborhoods and HIV: a social ecological approach to prevention and care. Am Psychol.

[5] Black D, Gates G, Sanders S, Taylor L (2002). Why do gay men live in San Francisco?. J Urban Econ.

[6] Ghaziani A (2014). There Goes the Gayborhood?.

[7] Gudelunas D (2012). There's an app for that: the uses and gratifications of online social networks for gay men. Sex Cult.

[8] Tudor M Cyberqueer techno-practices: digital space-making and networking among Swedish gay men [doctoral dissertation].

[9] Simon Rosser BR, West W, Weinmeyer R (2008). Are gay communities dying or just in transition? Results from an international consultation examining possible structural change in gay communities. AIDS Care.

[10] Horvath KJ, Simon Rosser BR, Remafedi G (2008). Sexual risk taking among young internet-using men who have sex with men. Am J Public Health.

[11] Silverman B (1986). Density Estimation for Statistics and Data Analysis.

[12] Gueler A, Schoeni-Affolter F, Moser A, Bertisch B, Bucher HC, Calmy A, Cavassini M, Ledergerber B, Wandeler G, Egger M, Swiss HIV Cohort Study‚ Swiss National Cohort (2015). Neighbourhood socio-economic position, late presentation and outcomes in people living with HIV in Switzerland. AIDS.

[13] Burke-Miller JK, Weber K, Cohn SE, Hershow RC, Sha BE, French AL, Cohen MH (2016). Neighborhood community characteristics associated with HIV disease outcomes in a cohort of urban women living with HIV. AIDS Care.

[14] Hull MW, Montaner JSG (2013). HIV treatment as prevention: the key to an AIDS-free generation. J Food Drug Anal.

[15] Lourenço L, Colley G, Nosyk B, Shopin D, Montaner JSG, Lima VD, STOP HIV/AIDS Study Group (2014). High levels of heterogeneity in the HIV cascade of care across different population subgroups in British Columbia, Canada. PLoS One.

[16] Montaner JSG, Lima VD, Harrigan PR, Lourenço L, Yip B, Nosyk B, Wood E, Kerr T, Shannon K, Moore D, Hogg RS, Barrios R, Gilbert M, Krajden M, Gustafson R, Daly P, Kendall P (2014). Expansion of HAART coverage is associated with sustained decreases in HIV/AIDS morbidity, mortality and HIV transmission: the “HIV Treatment as Prevention” experience in a Canadian setting. PLoS One.

[17] (2014). 90-90-90—an ambitious treatment target to help end the AIDS epidemic.

[18] Badal HJ, Stryker JE, DeLuca N, Purcell DW (2018). Swipe right: dating website and app use among men who have sex with men. AIDS Behav.

[19] Roth Y (2016). Zero feet away: the digital geography of gay social media. J Homosex.

[20] Zwitter A (2014). Big Data ethics. Big Data Soc.

[21] Goedel WC, Duncan DT (2015). Geosocial networking app usage patterns of gay, bisexual, and other men who have sex with men: survey among users of Grindr, a mobile dating app. JMIR Public Health Surveill.

[22] Card KG, Lachowsky NJ, Cui Z, Shurgold S, Gislason M, Forrest JI, Rich AJ, Moore D, Roth E, Hogg RS (2017). Exploring the role of sex-seeking apps and websites in the social and sexual lives of gay, bisexual and other men who have sex with men: a cross-sectional study. Sex Health.

[23] Burrell ER, Pines HA, Robbie E, Coleman L, Murphy RD, Hess KL, Anton P, Gorbach PM (2012). Use of the location-based social networking application GRINDR as a recruitment tool in rectal microbicide development research. AIDS Behav.

[24] Kakietek J, Sullivan PS, Heffelfinger JD (2011). You've got male: internet use, rural residence, and risky sex in men who have sex with men recruited in 12 U.S. cities. AIDS Educ Prev.

[25] Downing MJ, Schrimshaw EW (2014). Self-presentation, desired partner characteristics, and sexual behavior preferences in online personal advertisements of men seeking non-gay-identified men. Psychol Sex Orientat Gend Divers.

[26] Gabert R, Thomson B, Gakidou E, Roth G (2016). Identifying high-risk neighborhoods using electronic medical records: a population-based approach for targeting diabetes prevention and treatment interventions. PLoS One.

[27] Ramjee G, Wand H (2014). Geographical clustering of high risk sexual behaviors in “hot-spots” for HIV and sexually transmitted infections in Kwazulu-Natal, South Africa. AIDS Behav.

[28] Kelly BC, Carpiano RM, Easterbrook A, Parsons JT (2012). Sex and the community: the implications of neighbourhoods and social networks for sexual risk behaviours among urban gay men. Sociol Health Illn.

[29] Kerr JC, Valois RF, Siddiqi A, Vanable P, Carey MP (2014). Neighborhood condition and geographic locale in assessing HIV/STI risk among African American adolescents. AIDS Behav.

[30] Buttram ME, Kurtz SP (2013). Risk and protective factors associated with gay neighborhood residence. Am J Mens Health.

[31] Nash CJ, Gorman-Murray A (2014). LGBT neighbourhoods and “new mobilities”: towards understanding transformations in sexual and gendered urban landscapes. Int J Urban Reg Res.

[32] Vaughan AS, Kramer MR, Cooper HLF, Rosenberg ES, Sullivan PS (2017). Activity spaces of men who have sex with men: an initial exploration of geographic variation in locations of routine, potential sexual risk, and prevention behaviors. Soc Sci Med.

[33] (2015). Statistics Canada.

[34] Tarrant C, Wobi F, Angell E (2013). Tackling health inequalities: socio-demographic data could play a bigger role. Fam Pract.

[35] Chen J, Vargas-Bustamante A, Mortensen K, Ortega AN (2016). Racial and ethnic disparities in health care access and utilization under the Affordable Care Act. Med Care.

[36] Wong A, Wouterse B, Slobbe LCJ, Boshuizen HC, Polder JJ (2012). Medical innovation and age-specific trends in health care utilization: findings and implications. Soc Sci Med.

[37] Filc D, Davidovich N, Novack L, Balicer RD (2014). Is socioeconomic status associated with utilization of health care services in a single-payer universal health care system?. Int J Equity Health.

[38] Derose KP, Escarce JJ, Lurie N (2007). Immigrants and health care: sources of vulnerability. Health Aff (Millwood).

[39] Tiagi R (2016). Access to and utilization of health care services among Canada's immigrants. Intl J Migration Health Soc Care.

[40] Umberson D, Montez JK (2010). Social relationships and health: a flashpoint for health policy. J Health Soc Behav.

[41] Hallisey E, Tai E, Berens A, Wilt G, Peipins L, Lewis B, Graham S, Flanagan B, Lunsford NB (2017). Transforming geographic scale: a comparison of combined population and areal weighting to other interpolation methods. Int J Health Geogr.

[42] Dohoo IR, Martin SW, Stryhn H (2012). Methods in Epidemiologic Research.

[43] Kraemer W, Sonnberger H (2012). The Linear Regression Model Under Test.

[44] Cook R, Weisberg S (1982). Residuals and Influence in Regression.

[45] Tikkanen R, Ross MW (2003). Technological tearoom trade: characteristics of Swedish men visiting gay Internet chat rooms. AIDS Educ Prev.

[46] Card K, Lachowsky N, Cui Z, Shurgold S, Gislason M, Forrest J, Rich A, Moore D, Roth E, Hogg R (2016). Exploring the role of sex-seeking apps and websites in the social and sexual lives of gay, bisexual and other men who have sex with men: a cross-sectional study. Sex Health.

[47] Williams ML, Bowen AM, Horvath KJ (2005). The social/sexual environment of gay men residing in a rural frontier state: implications for the development of HIV prevention programs. J Rural Health.

[48] Wimark T (2014). Beyond Bright City Lights: The Migration Patterns of Gay Men and Lesbians.

[49] Van de Wiele C, Tong S (2014). Breaking boundaries: the uses & gratifications of Grindr.

[50] Crooks R (2013). The Rainbow Flag and the Green Carnation: Grindr in The Gay Village.

[51] Rice E, Holloway I, Winetrobe H, Rhoades H, Barman-Adhikari A, Gibbs J, Carranza A, Dunlap D (2012). Sex risk among young men who have sex with men who use Grindr, a smartphone geosocial networking application. J AIDS Clin Res.

[52] Annes A, Redlin M (2012). Coming out and coming back: rural gay migration and the city. J Rural Stud.

[53] Gorman-Murray A (2007). Rethinking queer migration through the body. Soc Cult Geogr.

[54] Grov C, Breslow AS, Newcomb ME, Rosenberger JG, Bauermeister JA (2014). Gay and bisexual men's use of the Internet: research from the 1990s through 2013. J Sex Res.

[55] Allman D, Meyers T, Xu K, Steele S (2012). The social technographics of gay men and other men who have sex with men (MSM) in Canada: implications for HIV research, outreach and prevention. Digit Cult Educ.

[56] Pearson J, Wilkinson L (2017). Same-sex sexuality and educational attainment: the pathway to college. J Homosex.

[57] Zhang X (2015). Low income measurement in Canada: what do different lines and indexes tell us?.

[58] Giles P (2004). Low income measurement in Canada.

[59] Bauer GR (2014). Incorporating intersectionality theory into population health research methodology: challenges and the potential to advance health equity. Soc Sci Med.

[60] Dhoest A, Szulc L (2016). Navigating online selves: social, cultural, and material contexts of social media use by diasporic gay men. Soc Media Soc.

[61] O'Neill H, Kia B (2012). Cent Excell Res Immigr Divers.

[62] Dalgleish J, Porter E (2016). Inclusivity and othering in Montréal's gay village.

[63] Barrett DC, Pollack LM (2016). Whose gay community? Social class, sexual self-expression, and gay community involvement. Sociol Q.

[64] Lipsitz MC, Segura ER, Castro JL, Smith E, Medrano C, Clark JL, Lake JE, Cabello R (2014). Bringing testing to the people—benefits of mobile unit HIV/syphilis testing in Lima, Peru, 2007-2009. Int J STD AIDS.

[65] Gilbert M, Salway T, Haag D, Fairley CK, Wong J, Grennan T, Uddin Z, Buchner CS, Wong T, Krajden M, Tyndall M, Shoveller J, Ogilvie G (2017). Use of GetCheckedOnline, a comprehensive Web-based testing service for sexually transmitted and blood-borne infections. J Med Internet Res.

[66] Fitzpatrick K, LaGory M (2002). Unhealthy Places: The Ecology of Risk in the Urban Landscape.

